# Remdesivir therapy in patients with COVID-19: A systematic review and meta-analysis of randomized controlled trials

**DOI:** 10.1016/j.amsu.2020.12.051

**Published:** 2021-01-06

**Authors:** Charan Thej Reddy Vegivinti, John M. Pederson, Kavitha Saravu, Nitin Gupta, Averi Barrett, Amber R. Davis, Kevin M. Kallmes, Kirk W. Evanson

**Affiliations:** a Department of General Medicine, Kasturba Medical College, Manipal Academy of Higher Education (MAHE), Manipal, Karnataka, India; b Nested Knowledge, St. Paul, MN, USA; c Department of Infectious Diseases, Kasturba Medical College, Manipal Academy of Higher Education (MAHE), Manipal, Karnataka, India; d Superior Medical Experts, St. Paul, MN, USA

**Keywords:** Coronavirus, SARS virus, Antiviral agents, Therapeutic uses

## Abstract

**Purpose:**

To perform a systematic review and meta-analysis of randomized controlled trials that examined remdesivir treatment for COVID-19.

**Materials and methods:**

A systematic literature search was performed using Pubmed, Embase, and ClinicalTrials.gov to identify studies published up to October 25, 2020 that examined COVID-19 treatment with remdesivir. A total of 3 randomized controlled trials that consisted of 1691 patients were included in the meta-analysis.

**Results:**

The odds for mechanical ventilation (MV) or extracorporeal membrane oxygenation (ECMO) following treatment was significantly lower in the remdesivir group compared to the control group (OR = 0.48 [95% CI: 0.34; 0.69], *p* < 0.001). The odds of early (at day 14/15; OR = 1.42 [95% CI: 1.16; 1.74], *p* < 0.001) and late (at day 28/29; OR = 1.44 [95% CI: 1.16; 1.79], *p* = 0.001) hospital discharge were significantly higher in the remdesivir group compared to the control group. There was no difference in the odds for mortality in patients treated with remdesivir (OR = 0.77 [95% CI: 0.56; 1.06], *p* = 0.108).

**Conclusions:**

Remdesivir attenuates disease progression, leading to lower odds of MV/ECMO and greater odds of hospital discharge for COVID-19 patients. However, remdesivir does not affect odds of mortality.

## Highlights

• Remdesivir reduces the odds for mechanical ventilation or extracorporeal membrane oxygenation in COVID-19 patients.
• Remdesivir improves the odds for hospital discharge.
• Remdesivir does not lower the odds for mortality.
• Remdesivir can attenuate COVID-19 progression, but it is less effective in treating critically-ill patients.

## 1 Introduction

There have been approximately 65.8 million confirmed cases of coronavirus disease (COVID-19) as of December 7, 2020, which has led to approximately 1.5 million deaths worldwide [1]. COVID-19, the disease caused by severe acute respiratory syndrome coronavirus 2 (SARS-CoV-2), can lead to acute respiratory distress syndrome (ARDS), primarily in immunocompromised patients, the elderly, and individuals with comorbidities (e.g., obesity, hypertension, chronic obstructive pulmonary disease) [[2], [3], [4]]. While current therapies aim to prevent respiratory complications, effective pharmacological therapies that target the virus are lacking.

Remdesivir, an adenosine nucleotide analog that inhibits SARS-CoV-2 RNA-dependent RNA polymerase [5], was recently approved for COVID-19 treatment in adults and pediatric patients (≥12 years) by the U.S. Food & Drug Administration [6]. However, the generalized treatment effects of remdesivir across multiple clinical outcomes and populations are poorly understood due to the small number of available studies, which includes studies that were terminated early due to a lack of COVID-19 patients [7]. Here, we performed a systematic review and meta-analysis in an effort to pool results from randomized controlled trials (RCTs) to better characterize the efficacy of remdesivir for the treatment of COVID-19.

## 2 Methods

A systematic literature search was performed to identify studies that examined COVID-19 treatment with remdesivir. Search terms included the following: “(remdesivir OR GS-5734) AND (COVID-19 OR SARS-CoV-2)”. Literature searches were performed in PubMed, Embase, and ClinicalTrials.gov up to October 25, 2020. The following article types were excluded: meta-analysis or review, editorial, opinion article, correspondence, letter to the editor, technical note, *in vitro* or *in vivo* study, methods article, protocol, case report, recommendations, or guidelines. Studies were also excluded if they failed to report remdesivir as a COVID-19 treatment, if they did not report patient outcomes, or if they only possessed one arm (no comparison group). Risk of bias and levels of evidence for each study was assessed as described in **Supplemental Methods**. Primary outcomes were need for mechanical ventilation (MV) or extracorporeal membrane oxygenation (ECMO), hospital discharge (early and late), and mortality.

### 2.1 Data analysis

All data were entered into a Microsoft Excel sheet and imported to R for analysis using the metafor package [8]. The ‘digitize’ package was used to extract data directly from figures in some cases [9]. We used Higgin's *I*^2^ statistics to estimate the percentage of variability in effect estimates that is due to heterogeneity rather than sampling error [10]. Effect sizes were computed as log transformed odds ratios (ORs) using the exact Mantel-Haenszel method [11]. To aid in interpretation, log transformed effect sizes were converted to a probability scale. A separate random effects model was fit for each outcome measure. Accordingly, the between-study variance component was estimated using a restricted effects maximum likelihood (REML) estimator with 95% CIs computed using the Q-profile method [12]. All statistical analyses were performed in RStudio (Version 1.3.959, RStudio, PBC).

## 3 Results

**Fig. 1:**
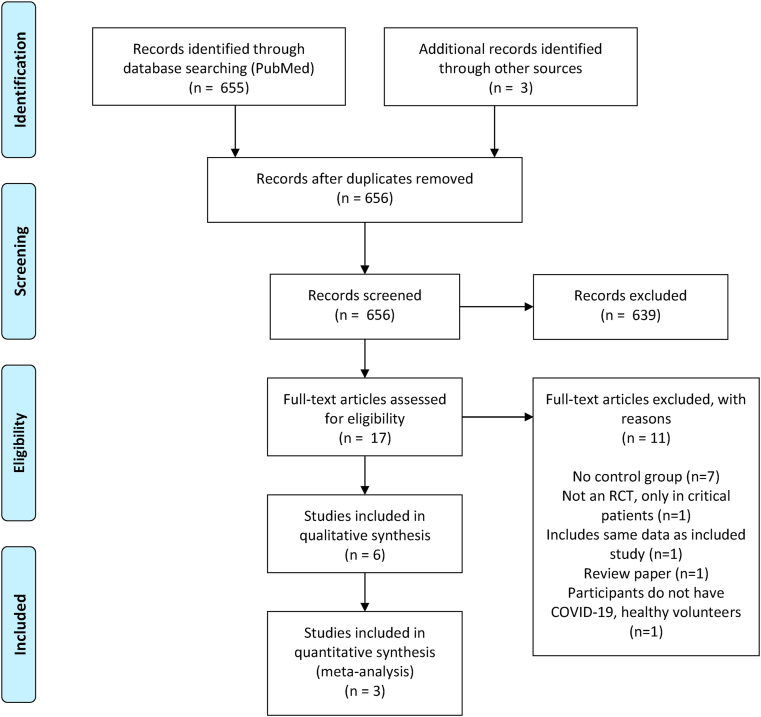
PRISMA diagram of search records and included studies.

A total of 655 articles were screened that fulfilled search criteria, of which 17 articles were selected for full-text review (Fig. 1). Three randomized controlled trials (RCTs) with a total study population of 1691 patients were included in the quantitative meta-analysis [[7], [13], [14]]. Among this patient population, 892 (52.7%) patients received remdesivir for 10 days and 799 (47.3%) patients received control therapies. Remdesivir was administered as a 200 mg loading dose on day 1, followed by 100 mg doses daily until the end of treatment. While remdesivir was administered over 10 days for all studies, Spinner et al. included a 5-day regimen in addition to the 10-day regimen. Baseline characteristics of the studies included in the meta-analysis are provided in Table 1.

**Table 1:** Study characteristics at baseline and primary conclusion.

Author	Arm	Age	NIV/HF n/N (%)	MV/ECMO n/N (%)	Conclusion
Beigel et al. [14]	Control	59.2 ± 15.4	98/521 (18.8%)	154/521 (29.6%)	RDV reduced the time to recovery in COVID-19 patients.
	RDV	58.6 ± 14.6	95/541 (17.6%)	131/541 (24.2%)	
Spinner et al. [13]	Control	57 (45–66)	2/200 (1%)	0/200 (0%)	RDV (5D) improved clinical status in COVID-19 patients as compared to control at day 11.
	RDV (5D)	58 (48–66)	2/191 (1%)	0/191 (0%)	
	RDV (10D)	56 (45–66)	1/193 (1)	0/193 (0%)	
Wang et al. [7]	Control	64 (53–70)	9/78 (12%)	1/78 (1%)	RDV did not improve clinical status of COVID-19 patients
	RDV	66 (57–73)	28/158 (18%)	0/158 (0%)	

Age is expressed as mean ± standard deviation or median (interquartile range). RDV = remdesivir; NIV = noninvasive ventilation; HF = high flow oxygen; MV = mechanical ventilation; ECMO = extracorporeal membrane oxygenation; 5D = 5 day; 10D = 10 day.

### 3.1 Need for mechanical ventilation or extracorporeal membrane oxygenation

**Fig. 2:**
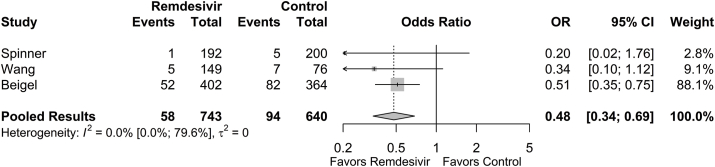
**Forest plot of subgroup comparisons of need for mechanical ventilation or ECMO at 28/29 days.** Pooled results were computed using restricted effects maximum likelihood with 95% confidence intervals computed using the Q-profile method. A 95% prediction interval was also computed (black bar).

Cumulative rates of MV or ECMO over the 28-day or 29-day duration of studies were collected. At final follow-up, the proportion of patients requiring MV or ECMO was 0.036 (95% CI: 0.006; 0.181) in the remdesivir group and 0.088 (95% CI: 0.024; 0.277) in the control group. The need for MV or ECMO was significantly lower in the remdesivir group compared to the control group (OR = 0.48 [95% CI: 0.34; 0.69], *p* < 0.001; Fig. 2). The estimated between-study variability unattributable to sampling error ranged from low to high (*I*^2^ = 0.0% [95% CI: 0.0%; 79.6%]).

### 3.2 Hospital discharge

**Fig. 3:**
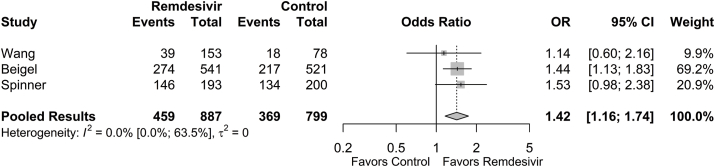
**Forest plot of subgroup comparisons of hospital discharge at 14/15 days.** Pooled results were computed using restricted effects maximum likelihood with 95% confidence intervals computed using the Q-profile method. A 95% prediction interval was also computed (black bar).

Cumulative rates of hospital discharge at 14 or 15 days (early discharge) as well as at 28 or 29 days (final follow-up) were collected. The proportion of patients achieving early hospital discharge was 0.508 (95% CI: 0.230; 0.781) in the remdesivir group and 0.435 (95% CI: 0.209; 0.692) in the control group. The odds of early hospital discharge were significantly higher in the remdesivir group compared to the control group (OR = 1.42 [95% CI: 1.16; 1.74], *p* < 0.001; Fig. 3). The estimated between-study variability unattributable to sampling error ranged from low to moderate (*I*^2^ = 0.0% [95% CI: 0.0%; 63.5%]).

**Fig. 4:**
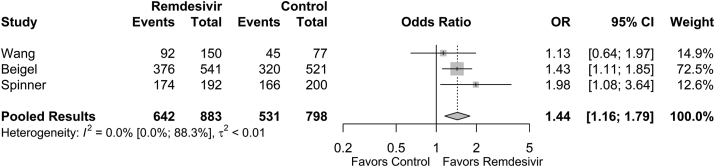
**Forest plot of subgroup comparisons of hospital discharge at 28/29 days.** Pooled results were computed using restricted effects maximum likelihood with 95% confidence intervals computed using the Q-profile method. A 95% prediction interval was also computed (black bar).

The proportion of patients achieving hospital discharge at final follow-up was 0.763 (95% CI: 0.526; 0.903) in the remdesivir group and 0.689 (95% CI: 0.507; 0.827) in the control group. The odds of hospital discharge at final follow-up was significantly higher in the remdesivir group compared to the control group (OR = 1.44 [95% CI: 1.16; 1.79], *p* = 0.001; Fig. 4). The estimated between-study variability unattributable to sampling error ranged from low to high (*I*^2^ = 0.0% [95% CI: 0.0%; 88.3%]).

### 3.3 Mortality

**Fig. 5:**
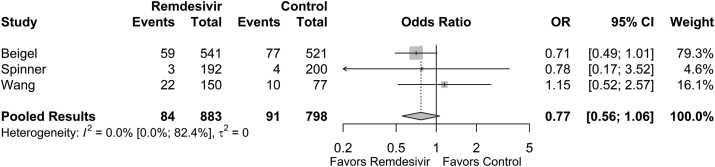
**Forest plot of subgroup comparisons of mortality at 28/29 days.** Pooled results were computed using restricted effects maximum likelihood with 95% confidence intervals computed using the Q-profile method. A 95% prediction interval was also computed (black bar).

Cumulative mortality rates at 28 or 29 days (final follow-up) were collected. Mortality rates at final follow-up were 0.071 (95% CI: 0.019; 0.228) in the remdesivir group and 0.080 (95% CI: 0.023; 0.239) in the control group. The odds of mortality between the remdesivir group and the control group were not significantly different (OR = 0.77 [95% CI: 0.56; 1.06], *p* = 0.108; Fig. 5). The estimated between-study variability unattributable to sampling error ranged from low to high (*I*^2^ = 0.0% [95% CI: 0.0%; 82.4%]).

### 3.4 Risk of bias

Of the RCTs included in the quantitative meta-analysis, 2 studies were considered high-quality (++) and 1 study was considered acceptable (+) according to the SIGN methodology for controlled trials. All studies demonstrated sufficient congruity between the research methodology, methods of data collection, study methodology, and interpretation of results and conclusions. As such, no studies were excluded based on quality. The results of our quality appraisal are summarized in Supplementary File 1.

## 4 Discussion

Here, we performed a systematic review and meta-analysis of studies that examined remdesivir treatment for COVID-19. COVID-19 patients that received remdesivir had lower odds for MV or ECMO following treatment as compared to patients that received control therapy. Remdesivir increased the odds for hospital discharge; however, remdesivir treatment did not reduce the odds for mortality in COVID-19 patients.

Remdesivir appeared to attenuate the progression of COVID-19 as evidenced by lower odds of MV or ECMO and greater odds for patient recovery (discharge). In the Adaptive COVID-19 Treatment Trial (ACTT-1), which consisted of 1062 patients with confirmed COVID-19 and evidence of lower respiratory tract involvement, 13% of patients treated with remdesivir (10 days) required MV or ECMO after treatment while 23% of control patients required that same level of support [14]. Fewer remdesivir patients (17%) required noninvasive ventilation (NIV) or high-flow oxygen as compared to control (24%). The need for MV or ECMO following treatment was also lower in the remdesivir groups of Spinner et al. (1% vs. 3%) [13] and Wang et al. (3% vs. 9%) [7], although the magnitude of treatment effects were smaller. In patients receiving MV or ECMO at enrollment, remdesivir treatment reduced the number of days on MV or ECMO as compared to placebo (17 vs. 20 days) [14]. Similarly, patients requiring any oxygen support at enrollment required fewer days of support with remdesivir (13 vs. 21 days) than placebo. These data indicate that remdesivir reduces the need for MV or ECMO and may provide some benefit to mitigate overall oxygen requirements in COVID-19 patients.

The odds for hospital discharge were greater with remdesivir treatment at 14/15- and 28/29-day time points. In the ACTT-1 trial, the median time to recovery was 50% longer in the control group (15 days) as compared to patients that received remdesivir (10 days) [14], and Wang et al. reported a similar treatment effect (control: 23 days; remdesivir: 18 days) [7]. Moreover, patients who received remdesivir were more likely to experience improvement in clinical status as compared to placebo [14] or standard therapy [13]. Time to recovery favored remdesivir in patients who required supplementary oxygen but were not critically ill [14]. Indeed, remdesivir treatment was less effective at expediting recovery in patients with greater disease severity. Beigel et al. noted that the risk ratios for time to recovery did not favor remdesivir in patients that required NIV/high flow oxygen or MV/ECMO at baseline, although follow-up times may have been insufficient for definitive conclusions [14]. The median time from onset of symptoms to start of treatment ranged from 9 to 10 days [7,13,14]; however, Beigel et al. noted that the rate ratio for recovery decreased from 1.37 (1.14–1.64) to 1.20 (0.94–1.52) in patients treated after 10 days from onset of symptoms. In a patient with COVID-19, the viremic phase lasts for a few days, which can then be followed by a hyper-inflammatory response. Early treatment may attenuate viremia; and therefore, blunt the hyper-inflammatory response as well. This may explain why remdesivir works in patients with early illness but not in those where the hyper-inflammatory response has already set in. These data suggest that remdesivir is effective at mitigating disease progression and can expedite recovery in patients, especially when administered early during the disease course. However, remdesivir may be less effective at enhancing recovery times in critically ill patients.

Remdesivir did not lower the odds for mortality in COVID-19 patients. However, disease severity and age differed across studies (Table 1), which could have influenced these results. Total mortality data from Spinner et al. were the lowest observed in the present analysis, which was consistent with the moderate level of disease reported in this study [13]. Indeed, <1% of COVID-19 patients in this study required NIV/high-flow oxygen at baseline and no patients required MV/ECMO. Thus, the moderate severity of disease overall would be associated with a relatively lower odds for mortality, which would make it difficult to see a meaningful reduction in mortality odds with remdesivir. Wang et al. detected similar rates of mortality between remdesivir (14%) and placebo (13%) [7], while Beigel et al. noted lower, albeit nonsignificant, mortality rates with remdesivir (7% vs. 12%) [14]. Wang et al. had fewer total patients (16%) that required NIV/high-flow oxygen or MV/ECMO as compared to Beigel et al. (45%); however, the patient population was older (Table 1), which could have contributed to similar overall mortality despite lesser disease severity as observed in Beigel et al. As discussed previously, the absence of a mortality benefit could be due, in part, to remdesivir's inability to rescue clinical deterioration in critically ill patients. In Beigel et al. mortality analysis by clinical status favored remdesivir in patients with a category 5 status (hospitalized, requiring supplemental oxygen; HR: 0.30 [0.14–0.64]), while no benefit was observed in patients with category 6 (hospitalized, requiring NIV or high-flow oxygen; HR: 1.02 [0.54–1.91]) or 7 status (hospitalized, requiring MV or ECMO; HR: 1.13 [0.67–1.89]). Although Wang et al. failed to detect a significant effect of remdesivir on mortality, the authors did note a statistically insignificant shift in mortality as related to early remdesivir treatment. Patients that received remdesivir within 10 days from symptom onset exhibited lower rates of mortality (11% vs. 15%). In contrast, patients that received remdesivir >10 days from symptom onset exhibited higher rates of mortality (14% vs. 10%) as compared to placebo. In all, these data suggest that remdesivir does not lower the odds of mortality in COVID-19 patients, potentially due to its inability to rescue clinical deterioration of critically-ill patients.

We did not include clinical improvement in our meta-analysis due to the heterogeneity of methods used to determine clinical improvement in the included studies. Clinical status scales, while possessing similar qualities, differed in the number of clinical categories (6–8), the directionality of the scale (category 1: discharged [7,14] vs. dead [13]), and the methods used to determine clinical improvement could vary according to the magnitude of improvement achieved per patient (+1, +2). Nevertheless, 2 of the 3 randomized controlled trials detected significant improvements in clinical status [13,14], while Wang et al. was underpowered due to early termination of the study [7]. Patients that received remdesivir experienced clinical improvement with odds ratios that ranged from 1.60 to 1.65 in favor of remdesivir [13,14]. Clinical improvement was achieved with 5- and 10-day regimens and was assessed 4–5 days following the end of treatment. Spinner et al. did not report statistically significant clinical improvement with their 10-day remdesivir arm (assessed at day 11, p = 0.18), although this study was open-label and it was believed that this design had some effect on clinical outcomes [13]. It was noted that by day 14, patients that received either 5- or 10-day remdesivir treatments exhibited improvements in clinical status as compared to standard therapy. While Spinner et al. noted clinical improvement, the meaningfulness of improvement was uncertain (e.g., category 7 [not hospitalized]: 76% remdesivir vs. 67% standard therapy).

Several single and double (multiple remdesivir dosage regimens, no comparator) arm studies have noted improvements in clinical status with remdesivir, especially in patients with non-critical forms of disease [15,16]. Antinori et al. (2020) noted better clinical outcomes (7-point ordinal scale) with remdesivir in non-ICU patients [16]. At 28 days, only 33% of ICU patients treated with remdesivir had been discharged as compared to 82% of non-ICU patients. Gilead Sciences conducted a randomized, open-label trial that consisted of 397 severe COVID-19 patients treated with remdesivir, of which 200 patients were treated for 5 days and 197 patients were treated for 10 days [17]. It is important to note that only 2% of patients in the 5-day group and 5% of patients in the 10-day group required MV or ECMO at baseline. The study demonstrated similar improvement (2 points or more on an ordinal scale) in clinical status in both groups on Day 14 (5-day: 64%, 10-day: 54%), after adjusting for differences in baseline clinical status. Taken together, these data suggest that remdesivir treatment improves clinical status of COVID-19 patients, especially in patients with non-critical forms of disease. However, the magnitude of improvement in clinical status is moderate.

### 4.1 Limitations

There were a limited number of randomized controlled studies to assess the efficacy of remdesivir treatment for COVID-19. Given the moderate but significant treatment effect, we decided to exclude small observational studies to reduce the statistical noise and underlying bias these studies can potentially contribute to the analysis in order to better characterize treatment effects associated with remdesivir.

## 5 Conclusions

Remdesivir treatment reduced the need for MV and improved hospital discharge rates. However, a mortality benefit with remdesivir is unclear. Ongoing clinical trials will further elucidate remdesivir's role as a COVID-19 therapy.

## Declaration of competing interest

The authors declare no interests with the subject of this manuscript. J.M.P. is employed by Nested Knowledge, Superior Medical Experts, and Marblehead Medical. K.M.K. works for and holds equity in Nested Knowledge, Superior Medical Experts, and Marblehead Medical. A.R.D. and K.W.E. are employed by Superior Medical Experts.

## Registration of research studies

Not applicable.

Name of the registry:

Unique Identifying number or registration ID:

Hyperlink to your specific registration (must be publicly accessible and will be checked):
