# Supplementary material for: Remdesivir therapy in patients with COVID-19: A systematic review and meta-analysis of randomized controlled trials
Source: Ann Med Surg (Lond). 2021 Jan 6;62:43–8. doi: 10.1016/j.amsu.2020.12.051 (PMC7806502; doi:10.1016/j.amsu.2020.12.051)
Supplement: Multimedia component 2 [file mmc2.docx]

**DATA SUPPLEMENT**

**SUPPLEMENTAL METHODS**

***Risk of bias assessment***

The risk of bias and levels of evidence of each study was scored using the Scottish Intercollegiate Guidelines Network (SIGN) checklists for controlled clinical trials [1]. As such, individual items on checklists were categorized as follows:

- “Well addressed” or “Yes”
- “Adequately addressed”
- “Poorly addressed” or “No”
- “Not reported”
- “Not applicable (N/A)”

The risk of bias among individual studies was coded as follows:

- High quality (++)
- Acceptable quality (+)
- Low quality (-)
- Unacceptable (0)

The risk of bias assessment was completed independently by 2 authors (AB and MS). Any disagreements were discussed and resolved by a third author (JMP).

**SUPPLEMENTAL REFERENCES**

[1] SIGN 50: A guideline developer's handbook, Scottish Intercollegiate Guidelines Network, 2011.
